# Non-Invasive Brain Stimulation in Neglect Rehabilitation: An Update

**DOI:** 10.3389/fnhum.2013.00248

**Published:** 2013-06-10

**Authors:** René Martin Müri, Dario Cazzoli, Tobias Nef, Urs P. Mosimann, Simone Hopfner, Thomas Nyffeler

**Affiliations:** ^1^Division of Cognitive and Restorative Neurology, Departments of Neurology and Clinical Research, Inselspital, Bern University Hospital, and University of Bern, Bern, Switzerland; ^2^Gerontechnology and Rehabilitation Research Group, ARTORG Center for Biomedical Engineering Research, University of Bern, Bern, Switzerland; ^3^Nuffield Department of Clinical Neurosciences, University of Oxford, Oxford, UK; ^4^Department of Internal Medicine, Center of Neurology and Neurorehabilitation, Luzerner Kantonsspital, Luzern, Switzerland

**Keywords:** review, rehabilitation, unilateral neglect, transcranial magnetic stimulation, theta-burst protocol, transcranial direct current stimulation

## Abstract

Here, we review the effects of non-invasive brain stimulation such as transcranial magnetic stimulation (TMS) or transcranial direct current stimulation (tDCS) in the rehabilitation of neglect. We found 12 studies including 172 patients (10 TMS studies and 2 tDCS studies) fulfilling our search criteria. Activity of daily living measures such as the Barthel Index or, more specifically for neglect, the Catherine Bergego Scale were the outcome measure in three studies. Five studies were randomized controlled trials with a follow-up time after intervention of up to 6 weeks. One TMS study fulfilled criteria for Class I and one for Class III evidence. The studies are heterogeneous concerning their methodology, outcome measures, and stimulation parameters making firm comparisons and conclusions difficult. Overall, there are however promising results for theta-burst stimulation, suggesting that TMS is a powerful add-on therapy in the rehabilitation of neglect patients.

## Introduction

Hemispatial neglect is a common neurological syndrome that may be particularly disabling after stroke. It is defined as the failure to detect, respond, or orient to the stimuli located in the portion of space contralateral to the lesion (Heilman et al., 1993). Neglect is common, occurring in up to 43% of patients suffering from an acute right-hemispheric stroke (Ringman et al., 2004). Depending on the assessment, the reported incidence may widely vary between 10 and 82% following right-hemispheric damage and between 15 and 65% following left-hemispheric damage (Plummer et al., 2003). Neglect patients show slower functional progress during rehabilitation and need longer hospitalization (Cherney et al., 2001; Gillen et al., 2005). Furthermore, neglect is an independent predictor of poor outcome, in terms of more limited functional independence (Stone et al., 1992; Di Monaco et al., 2011) and lower likelihood of being discharged home (Wee and Hopman, 2005, 2008).

Different therapeutic strategies to treat neglect have been evaluated, such as visual scanning, prism adaptation, sensory stimulation, neck muscle vibration, optokinetic stimulation, or pharmacologic treatments (see for a review Bowen et al., 2002; Kerkhoff and Schenk, 2012). Although these treatments attenuate the severity of neglect, they are often difficult to apply in rehabilitation – particularly during the acute or subacute phase of stroke – due to short duration of effects, patient discomfort, or the difficulty for patients to cooperate (Fierro et al., 2006).

### The concept of interhemispheric rivalry in neglect

The concept of interhemispheric rivalry, based on the model by Kinsbourne (1987, 1993), is so far the most common basis for the application of non-invasive brain stimulation (NIBS) to modulate neglect [newer promising approaches are however also thinkable, such as, e.g., rhythmic transcranial magnetic stimulation (TMS) (see Thut et al., 2011) or network modulations (see van der Werf et al., 2010)]. According to this concept, both parietal cortices exert reciprocal interhemispheric inhibition. A damage of the right parietal cortex causes disinhibition of the intact, left hemisphere, and thus a pathological over-activation of the latter. This over-activation in the left, intact hemisphere further depresses the neural activity by an increased inhibition on the damaged hemisphere, aggravating the rightward, ipsilesional attentional bias.

Evidence supporting this concept comes from several experimental approaches. First, seminal works in animal models (Sprague, 1966) and a large body of subsequent studies (see, e.g., Payne and Rushmore, 2004; Rushmore et al., 2006; Valero-Cabré et al., 2006) showed that: (a) unilateral interventions (such as lesion, cooling, or TMS) generally introduce an imbalance in the physiological activity between the networks controlling visuospatial attention in the two hemispheres, favoring the intact hemisphere and leading to neglect; and (b) the experimental cancelation of this imbalance (and of neglect) is achievable through the reduction of the hyperexcitability (by lesion or cooling) of specific cortical or subcortical regions in the intact hemisphere. Second, fMRI studies showed a relative hyperactivity of the left, undamaged hemisphere in neglect patients, which correlated with neglect severity as measured by behavioral tasks (Corbetta et al., 2005). Moreover, the recovery of neglect correlated with the restoration and rebalancing of activity between both hemispheres, particularly in the dorsal parietal cortex (Corbetta et al., 2005; He et al., 2007). Third, clinical observations also indicate the relevance of the rebalancing of the activity between the two hemispheres as a functional mechanism accompanying neglect recovery. Vuilleumier et al. (1996) described the case of a patient who suffered from two sequential strokes. The first, right-hemispheric stroke, involving the parietal cortex, induced severe neglect, which completely recovered after a second, left-hemispheric stroke involving the frontal eye field. Fourth and finally, the pathological hyperactivity of intact, contralesional areas in neglect patients has also been directly demonstrated by means of a twin-coil TMS approach, allowing to assess the cortical excitability within parieto-motor circuits of the left hemisphere (Koch et al., 2008, 2012). Results showed a significantly higher excitability in neglect patients as compared to healthy controls and to patients with right-hemispheric lesions but no neglect. The degree of overexcitability was significantly correlated with neglect severity as measured by paper–pencil tests. Moreover, the application of inhibitory repetitive TMS (rTMS) over the left, contralesional posterior parietal cortex (PPC) could significantly reduce its overexcitability and triggered a significant amelioration in the behavioral measures of neglect.

The results illustrated above thus support the idea that the reinstatement of interhemispheric inhibitory balance is an important mechanism in neglect recovery.

### Non-invasive brain stimulation

Non-invasive brain stimulation, i.e., TMS or transcranial direct current stimulation (tDCS), has been increasingly used to interfere with brain activity in healthy subjects and patients with brain lesions. Depending on the stimulation parameters, it is possible to facilitate or to suppress brain activity with measurable behavioral effects.

Transcranial magnetic stimulation is based on the application of very short-lasting, strong electric currents delivered through a coil generating a rapidly changing, high-intensity magnetic field. This magnetic field induces on its part perpendicular currents in the brain, which are strong enough to directly depolarize neurons and influence cortical excitability. rTMS can either enhance (5–20 Hz, so-called high-frequency stimulation) or suppress (≤1 Hz, low-frequency stimulation) cortical activity and modulate excitability beyond the duration of the applied stimulation (see for a review Hallett, 2007).

More recently, the so-called “theta-burst stimulation” (TBS) has been introduced as a new protocol. Originally, such protocols were used to induce long-term potentiation (LTP) or long-term depression (LTD) in brain slices (Larson et al., 1986; Abraham, 2003). The protocol consists of three short trains of repetitive high-frequency TMS (30–100 Hz) in theta-frequency range (4–7 Hz). The stimulation pattern can have either excitatory (intermittent theta-burst, iTBS) or inhibitory (continuous theta-burst, cTBS) effects on brain activity (Huang et al., 2005). TMS can be used in a variety of ways to induce plastic changes in the brain. An effective way to modulate synaptic efficacy is to activate a cell with two or more inputs at brief intervals, such as in the bursts of the theta-burst protocol. A steady increase in synaptic strength is called LTP, a decrease LTD. In analogy, Huang et al. (2005) developed a modified TBS protocol with a pattern consisting of bursts of three pulses at 50 Hz, repeated every 200 ms intervals (i.e., at 5 Hz). The stimulation intensity was 80% of the activated motor threshold and the total number of pulses was 600. They found that a short and intermittent application of TBS (iTBS) facilitated motor-evoked potentials, i.e., increased their amplitude, whereas a continuous application of TBS (cTBS) suppressed motor-evoked potentials for up to 1 h. Nyffeler et al. (2006a, 2009) showed that such LTD-like effects could be disproportionately prolonged by repeated TBS application both in healthy subjects and in patients with neglect. They used a further modified theta-burst protocol with a burst frequency of 30 Hz, repeated with an interburst interval of 100 ms. The stimulation intensity was 80% of the resting motor threshold, and the total number of pulses was 801. The behavioral outcome was measured in healthy subjects with an oculomotor paradigm. The modified cTBS protocol has been shown to yield conspicuously longer inhibitory effects on the oculomotor cortex [i.e., the frontal eye field, in a head-to-head comparison with the commonly applied 1-Hz stimulation protocol (Nyffeler et al., 2006b)]. Moreover, Nyffeler et al. (2006a, 2009) showed that the behavioral effect of cTBS could be disproportionally prolonged: the behavioral effect after one, two, or four cTBS trains lasted on average up to 30 min, 3 h, or 11 h, respectively (Nyffeler et al., 2006a). Similar prolonged behavioral effects after repeated cTBS application were also found in patients with neglect. In a visual perception task, two cTBS trains significantly increased the number of perceived left visual targets for up to 8 h, whereas the application of four cTBS trains significantly increased the number of perceived left targets up to 32 h. No significant improvement was found after sham stimulation (Nyffeler et al., 2009).

While rTMS can generate strong currents capable to depolarize neurons, tDCS changes cortical activity by means of small electric currents. Suggested as a purely neuromodulatory approach, tDCS seems to alter brain activity by influencing the resting membrane potential, and does not evoke action potentials (Fregni and Pascual-Leone, 2007; Nitsche et al., 2008; Paulus, 2011). During tDCS, small currents (1–2 mA) are delivered to the brain transcranially via two large electrodes. The duration of the stimulation, its strength, and its polarity determine the excitability changes. Anodal tDCS leads to excitation of the brain, whereas cathodal tDCS results in brain inhibition (Nitsche and Paulus, 2000). tDCS effects seem to be mainly mediated by changes in the excitability of inhibitory or facilitatory interneuronal circuits that can outlast stimulation duration. tDCS has the advantage that the device is inexpensive, portable, and easy to use, in particular simultaneously with treatment sessions in the rehabilitation setting. Finally, the tingling sensation on the scalp at the beginning of the stimulation fades away shortly after. This is an advantage for a reliable sham condition (i.e., the device can be set to turn off a few seconds after the stimulation beginning, without the subject or the experimenter noticing it), and is also an important element for double-blind, controlled clinical trials.

The aim of the present study is to review the literature concerning the effectiveness of NIBS in the treatment of neglect patients.

## Methods

We searched the following databases for studies published in English: PubMed, PsychINFO, and Science Direct. Following search terms were used: neglect, visual neglect, unilateral neglect, rehabilitation, TMS, tDCS. Studies were included in the review if they satisfied following criteria: use of an offline TMS protocol, or use of an online or offline tDCS protocol; treatment of neglect or evaluation of the duration of NIBS effects on neglect as a goal of the study.

## Results

The characteristics of the included studies are presented in Tables 1 and 2. We found 10 studies that used TMS for neglect rehabilitation, and only 2 studies that used tDCS. In these studies, a total of 172 patients were involved, 147 patients in TMS studies and 25 patients in tDCS studies. The number of included patients varied considerably between studies, from 2 (Shindo et al., 2006) to 27 patients (Kim et al., 2013).

**Table 1:** **Studies evaluating treatment of neglect by TMS**.

Study	No. of patients	Time post	Sham control	Stimulation site (contra/ ipsilesional)	No. of pulses, frequency, intensity	No. of sessions	Time of assessment in relation to stimulation	Outcome measures	Main results	Descriptive magnitude of the changes in the main outcome measures
Brighina et al. (2003)	3 RH	3-5 m	No	Contra P5	900 Pulses, 1 Hz, 90% MT	7 sessions (every second d)	2 w/pre/post/2 w	Computerized length judgment task with prebisected lines. Clock drawing, line bisection	Sign. improvement in all tasks, at end and 2 w after stimulation	On average ∼ −0.6 pts (∼ −83%) at end and ∼ −0.57 pts (∼ −79%) at 2 w after stimulation in the mean scores of the computerized length judgment task (negative deflection = leftward bias)*; −4.6 mm (∼ −50%) between 2 w before and 2 w after stimulation in the mean rightward bias in the line bisection
Shindo et al. (2006)	2 RH	175, 186 d	No	Contra P5	900 pulses, 0.9Hz, 90% MT	6 sessions (3 per w)	2 w/1 d pre/1 d post/2 w/4 w/6 w	Two BIT subtests, MMSE, BRS, BI	Positive effects in BIT and BI for at least 6 w	Peak BIT-B of 38 pts and BIT-C of 100 pts after rTMS (pre = ∼20 and ∼60 pts*) in one patient, 35 and 83 pts (pre = ∼10 and 40 pts*) in the other
Koch et al. (2008)	10 RH, 5 RH without neglect	1–6 m	No	Contra P3	600 pulses, 1 Hz, 90% MT	1 session	Pre/post	MEP measures in the intact LH, naming test	Hyperexcitability of LH reduced after rTMS only in neglect patients. Sign. reduction of left-sided omissions	On average ∼ −40% in the MEP amplitude (% control, ISI of 4 ms)*; −13.9% in the left-sided omissions in the naming test
Song et al. (2009)	14 RH (7 treatment, 7 control)	15–60 d	No	Contra P3	450 pulses, 0.5Hz, 90% MT	14 sessions, 2 w (2 trains per d)	2 w/pre/post/2 w	Line bisection, line cancelation	Sign. improvement in both tasks in the rTMS group, up to 2 w after stimulation	Amelioration on average, according to the index values: ∼ −41% post and ∼ −38% at 2 w in the line bisection task; ∼ −79% post and ∼ −76% at 2 w in the line cancelation task*
Nyffeler et al. (2009)	11 RH	0.4–36.1 m	Yes	Contra P3	801 pulses cTBS, 30Hz, repeated at 100 ms, 100% MT	1 session (two or four cTBS trains)	Pre/1 h post/3 h/8 h/24 h/32 h/96 h	PVT	Sign. increase of detected left targets and reduction of RT only in the active cTBS condition. Stable effects up to 8 h after two cTBS trains, up to 32 h after four cTBS trains	On average: with two cTBS trains, from ∼8.1 omitted left targets pre to ∼3.5 at 8 h (≅ −57%); reaction times to left-sided targets from ∼6.9 s pre to ∼5.5 s at 8 h (≅ −21%). With four cTBS trains, from ∼7.1 omitted left targets pre to ∼1.7 at 32 h (≅ −76%); reaction times to left-sided targets from ∼7.4 s pre to ∼4.6 s at 32 h (≅ −38%)*
Kim et al. (2010)	19 RH	23.73 ± 12.3 m	Yes	Contra P3 and ipsi P4	1200 pulses, 1 Hz, 90% MT or 1000 pulses, 20Hz, 90% MT	1 session	Pre/post	Letter cancelation task, line bisection, Ota’s task	Sign. improvement in the Ota’s task after 1 Hz stimulation only	On average ∼ +1.6 responses to O in the left side, ∼ +1.85 correct responses to C in the left side, ∼ +1.8 correct responses to O in the left side as compared to sham in the Ota’s task (all mean sham values = 0)*
Lim et al. (2010)	7 RH, 7 controls	9–313 d	No	Contra P3	900 pulses, 1 Hz, 90% MT	10 sessions (1 session per d)	Pre/post	Line bisection test, Albert test	Sign. improvement in the line bisection test for left-sided line sets	On average 33.4% improvement in the line bisection test for left-sided line sets (median = 28.5%)
Koch et al. (2012)	9 RH, 9 controls	24–102 d	Yes	Contra P3	600 pulses cTBS at 50 Hz, repeated every 200 ms, 80% MT	10 sessions (two cTBS trains per d for 2 w)	Pre/post/2 w	MEP measures in the intact LH, BIT	Sign. improvement in the BIT after real stimulation up to 2 w. Hyperexcitability of LH reduced after rTMS in neglect patients only	On average ∼ −50% in the MEP amplitude (% control) after cTBS, ∼ −40% at 2 w*; 16.3% improvement of the BIT scores after cTBS, 22.6% at 2 w
Cazzoli et al. (2012)	16 RH, 8 RH control group	Mean 27 d (SEM 4.5 d)	Yes	Contra P3	801 pulses cTBS at 30Hz, repeated every 100 ms, 100%MT	2 sessions (four cTBS trains per d)	1 w pre/1 w post/2 w/3 w	PVT, CBS, random shape cancelation test, two part picture test, reading texts	Sign. improvement in all outcome measures only after real stimulation, at least for 3 w	On average 37% improvement in the spontaneous everyday behavior as measured by the CBS
Kim et al. (2013)	27 RH	Mean 15 d	Yes	Contra P3 and ipsi P4	1200 pulses, 1 Hz, 90% MT or 1000 pulses, 20Hz, 90% MT	10 sessions over 2 w (5 d per w)	Pre/post	Motor-free visual perception test, line bisection test, star cancelation test, CBS, K-MBI	Sign. improvement in the line bisection test after high-frequency rTMS and in the K-MBI after high and low rTMS	On average: -36.9% rightward deviation in the line bisection test after high-frequency stimulation (sham = −8.3%); +27.6 pts after low-frequency rTMS and +30.6 pts after high-frequency rTMS in the K-MBI scores (sham = +15.1 pts)

**Table 2:** **Studies evaluating treatment of neglect by tDCS**.

Study	No. of patients	Time post	Sham control	Stimulation site (contra/ipsilesional)	Protocol	No. of sessions	Time of assessment in relation to stimulation	Outcome measures	Main results	Descriptive magnitude of the changes in the main outcome measures
Ko et al. (2008)	15 RH	29–99 d	Yes	Ipsi P4	2.0 mA anodal stimulation for 20 min	1 session	Pre/post	Line bisection test, letter-structured cancelation test, shape-unstructured cancelation test	Sign. effects of real tDCS on line bisection test and shape-unstructured cancelation test	On average: −3.52 percent deviation score in the line bisection test (= ∼ −19%); −3.47 omissions in the shape-unstructured cancelation test (= ∼ −14.8%)
Sparing et al. (2009)	10 RH	0.5–12.4 m	Yes	Contra P3 and ipsi P4	1.0 mA anodal and cathodal stimulation for 10 min	2 sessions, cross-over	Pre/post	Line bisection test, visual detection task	Sign. improvement in line bisection test after anodal tDCS of the lesioned hemisphere and cathodal tDCS of the intact hemisphere	On average: in the line bisection test, from 3.4 mm deviation pre (rightwards bias) to -1.5 mm post (leftward bias) with anodal tDCS on P4; from 5.4 mm pre to −1.7 mm post with cathodal tDCS on P3

*w, week; m, month; d, day; MT motor threshold; MMSE, Mini Mental State Examination; BRS, Brunnstrom Recovery Index; BI, Barthel Index; PVT, subtest of the Vienna Test System (detection of peripheral visual targets); CBS, Catherine Bergego Scale; MEP, motor-evoked potential; RH, right hemisphere; LH, left hemisphere; RT, reaction time; K-MBI, Korean-Modified Barthel Index; SEM, standard error of the mean; pts, points*.

*These values have been visually inferred from the graphs provided in the respective studies.

The methodological differences in the rTMS protocols between the studies were also considerable. Five studies used low-frequency rTMS (Brighina et al., 2003; Shindo et al., 2006; Koch et al., 2008; Song et al., 2009; Lim et al., 2010), with frequencies of 0.5, 0.9, or 1 Hz. Three studies used cTBS (Nyffeler et al., 2009; Cazzoli et al., 2012; Koch et al., 2012) with either 30 or 50 Hz bursts. Finally, two studies (Kim et al., 2010, 2013) compared the effects of low-frequency (1 Hz) stimulation over the contralesional, intact hemisphere with those of high-frequency rTMS (20 Hz) over the ipsilesional hemisphere.

Further differences included the number of applied pulses, the duration of the intervention and of the observation period after the intervention, the type of coil used, and the procedure used to determine the stimulation location. The number of TMS pulses varied between 450 (Song et al., 2009) and 1200 pulses per session (Kim et al., 2010, 2013), the cumulative number was between 600 (Koch et al., 2008) and 12,600 pulses (Song et al., 2009). The intervention duration varied between a single session (Koch et al., 2008; Nyffeler et al., 2009; Kim et al., 2010) and 14 sessions (Song et al., 2009).

All studies used a focal, figure-of-eight coil, with the exception of Nyffeler et al. (2009) and Cazzoli et al. (2012), who used a round coil.

Concerning the location of the stimulation site, only one study used a neuronavigation system (Koch et al., 2012). They targeted the left PPC, using individual anatomic MRI and positioning the coil over the angular gyrus close to the posterior part of the adjoining intraparietal sulcus. All other studies used the international 10–20 EEG System. Two studies stimulated over P5 (Brighina et al., 2003; Shindo et al., 2006), all other studies over P3 (or, respectively, P4 for the two studies that entailed ipsilesional stimulation; Kim et al., 2010, 2013).

Five studies were sham-controlled (Nyffeler et al., 2009; Kim et al., 2010, 2013; Cazzoli et al., 2012; Koch et al., 2012), the remaining studies had no sham control group. A control group of patients without neglect was included in three studies (Koch et al., 2008; Song et al., 2009; Lim et al., 2010). One study (Koch et al., 2012) fulfilled the criteria for Class III evidence, one study (Cazzoli et al., 2012) the criteria for Class I evidence.

In only one study (Brighina et al., 2003) patients had no rehabilitation therapy during the observation. The patients in Lim’s study (Lim et al., 2010) received behavioral therapy, and the patients in Koch’s study (Koch et al., 2012) received 20 sessions of 45 min therapy. In the remaining four studies (Shindo et al., 2006; Song et al., 2009; Cazzoli et al., 2012; Kim et al., 2013), the patients received a full neurorehabilitation program, including occupational therapy, physiotherapy, and neuropsychology.

The time between brain damage and inclusion varied also considerably between studies. Patients in the acute/subacute stage (first 3 months after brain damage) were included in the studies by Song et al. (2009), Koch et al. (2012), Cazzoli et al. (2012), and Kim et al. (2013). Patients with chronic neglect (more than 3 months after brain damage) were included in the studies by Brighina et al. (2003), Shindo et al. (2006), and Kim et al. (2010). The remaining studies included both patients in the subacute or in the chronic stage.

The follow-up time of the observation of the stimulation effects ranged from 3 days (Nyffeler et al., 2009), 2 weeks (Brighina et al., 2003; Song et al., 2009; Koch et al., 2012), 3 weeks (Cazzoli et al., 2012) to 6 weeks (Shindo et al., 2006). In all studies, no information is provided about a potential fade-out of the stimulation effects over time.

## Discussion

Our database search resulted in 12 studies fulfilling the inclusion criteria. The studies are heterogeneous concerning methodology, evaluation, patients, and post-stroke inclusion time, making firm conclusions about the efficacy of NIBS difficult. In the last few years, at least five reviews (Fierro et al., 2006; Cazzoli et al., 2010; Hesse et al., 2011; Oliveri, 2011; Mylius et al., 2012) specifically addressed the application of TMS or tDCS for the treatment of neglect, and at least another 11 more general reviews (Dobkin, 2004; Rossi and Rossini, 2004; Miniussi et al., 2008; Schlaug and Renga, 2008; Marshall, 2009; Bashir et al., 2010; Langhorne et al., 2011; Miniussi and Rossini, 2011; Stuss, 2011; Vallar and Bolognini, 2011; Schulz et al., 2013) included the topic of brain stimulation in neglect. The number of reviews emphasizes the great interest in the development and establishment of new and current NIBS approaches for the treatment of neglect in particular, and for cognitive rehabilitation in general. However, the mismatch between the number of reviews and the number of original studies represents a compelling call for further systematic investigations in this field.

We found 10 studies using rTMS, and only 2 studies using tDCS. All rTMS studies used inhibitory protocols (low-frequency stimulation or cTBS) and stimulated the contralesional parietal cortex. Two studies (Kim et al., 2010, 2013) also included a condition in which the ipsilesional parietal cortex was stimulated using a high-frequency, excitatory protocol. Nine studies showed a significant improvement after inhibitory stimulation of the contralesional parietal cortex, one study (Kim et al., 2013) found a significant improvement only after ipsilesional excitatory stimulation. The number of patients included in the studies varied between 2 and 27 patients. Four studies evaluated only immediate effects after stimulation, without any follow-up measurements. The remaining six studies performed follow-up examinations up to 6 weeks. Five studies were not sham-controlled and, in three studies, activities of daily living (ADL) were evaluated in addition to neuropsychological testing. One study (Cazzoli et al., 2010) fulfilled Class I evidence, and one study (Koch et al., 2012) Class III evidence. In both studies, cTBS of the contralesional parietal cortex was applied.

To the best of our knowledge, no study so far directly compared the different forms of NIBS (e.g., TMS, tDCS) in order to demonstrate the superiority of one method. Both techniques present advantages and disadvantages, and the preference for the application of one technique or the other may also largely depend on the experimental questions and design (see Priori et al., 2009). Moreover, the application of TMS and tDCS should not be seen as mutually exclusive. The combination of the two techniques has in fact been shown to yield promising results, e.g., applying preconditioning by means of tDCS followed by rTMS application (Siebner et al., 2004).

In summary, notwithstanding the limited number of studies, the current state of the evidence looks more promising concerning the studies using cTBS. In the following, we will discuss methodological key points for the future development of treatment concepts of neglect by NIBS.

### Different effects of NIBS on outcome variables

In all studies, a battery of different neuropsychological tests, or test batteries specifically developed for neglect assessment (such as the behavioral inattention test, BIT) were used. Effects of stimulation were often strikingly different across outcome variables, suggesting possible dissociations. One explanation may be methodological: 8 out of the 10 rTMS studies used a focal figure-of-eight coil. Since neglect is associated with multiple lesion sites (e.g., Verdon et al., 2010; Corbetta and Shulman, 2011), a focal stimulation may not be sufficient to improve all aspects tapped by the different neuropsychological tests. It is noteworthy that Cazzoli et al. (2012), who used a non-focal round coil, found significant improvements in all tests. Thus, high focal precision may not be a primary goal for therapeutic rTMS application. However, further studies are needed to evaluate whether focal or non-focal rTMS stimulation of the network involved in neglect has a better clinical outcome.

Three studies also evaluated the effect of TMS on the ADL using the Barthel Index or the Catherine Bergego Scale. Shindo et al. (2006) used the Barthel Index and found a significant improvement after stimulation. Cazzoli et al. (2012) used the Catherine Bergego Scale and also found a significant improvement after real stimulation, but not after sham stimulation. Finally, Kim et al. (2013) used both the Barthel Index and the Catherine Bergego Scale and found a significant improvement only in the Barthel Index.

### Stimulation protocols

Generally, inhibitory stimulation protocols are predominantly applied. Low-frequency (0.5–1 Hz) repetitive stimulation was used in seven studies. The total number of pulses and daily application varied considerably between studies. The stimulation strength more consistently used was 90% of the motor threshold. Three studies used continuous inhibitory cTBS, one study (Koch et al., 2012) used the standard protocol described by Huang et al. (2005), two studies (Nyffeler et al., 2009; Cazzoli et al., 2012) the modified protocol described by Nyffeler et al. (2006a). The two protocols differ in the frequency of the bursts (50 versus 30 Hz), in the total number of pulses (600 versus 801 pulses), and in the definition of the stimulation strength (80% active motor threshold versus 100% resting motor threshold).

These two protocols were recently compared by Goldsworthy et al. (2012). They stimulated the human primary motor cortex in healthy subjects and recorded motor evoked-potentials (MEP) from the right first dorsal interosseous muscle before and at 0, 5, 10, 20, and 30 min after stimulation. The results showed that the standard protocol with 50 Hz induced a neuroplastic response that was short-lived and highly variable, whereas the modified protocol with 30 Hz induced a lasting change in MEP amplitude that was consistent between subjects. Such a lasting and consistent effect of cTBS may be an advantage for the therapeutic stimulation application. Furthermore, the fact that the repeated cTBS application at the same day can disproportionately prolong its effects (Nyffeler et al., 2009) is a further advantage.

### TBS – the way to an “ideal” stimulation protocol?

From a clinical and practical point of view, future stimulation protocols for therapeutical interventions should have the following properties: (1) the application should be easy to perform, i.e., no additional examinations such as neuroimaging or neuronavigation systems should be needed to localize the stimulation site. Indeed, only one study (Koch et al., 2012) used neuronavigation to localize the target site. The remaining studies localized the stimulation site by using the international 10–20 system. (2) The application time should be short. Protocols such as low-frequency stimulation protocols, with daily applications over several weeks, are difficult to perform in a rehabilitation clinic, and are often not well tolerated by patients. In contrast, cTBS application lasts about 40 s. Using the potential of disproportionate prolongation of the effects by repeated cTBS application, Cazzoli et al. (2012) could show that eight cTBS trains applied on 2 days have an ADL-relevant effect of up to 3 weeks.

## Conclusion

Our update and review of recent studies using NIBS for neglect treatment shows an ongoing evolution of TMS application from proof-of-concept studies to clinical application. However, the limited number of studies indicates the need of further systematic investigations in this field, with the aim of developing and establishing the most promising stimulation parameters. For cTBS, two recent Class I and III studies demonstrated its clinical utility as add-on therapy in neglect treatment. For tDCS application in neglect, only two studies were found, indicating that this technique may still be in an earlier stage in the evolution toward clinical application.

## Conflict of Interest Statement

The authors declare that the research was conducted in the absence of any commercial or financial relationships that could be construed as a potential conflict of interest.
